# Preclinical and Clinical Status of PSMA-Targeted Alpha Therapy for Metastatic Castration-Resistant Prostate Cancer

**DOI:** 10.3390/cancers13040779

**Published:** 2021-02-13

**Authors:** Asta Juzeniene, Vilde Yuli Stenberg, Øyvind Sverre Bruland, Roy Hartvig Larsen

**Affiliations:** 1 Department of Radiation Biology, Institute for Cancer Research, Norwegian Radium Hospital, Oslo University Hospital, Montebello, 0379 Oslo, Norway; vilde.stenberg@rr-research.no; 2 Nucligen, Ullernchausséen 64, 0379 Oslo, Norway; sciencons@gmail.com; 3 Institute for Clinical Medicine, University of Oslo, Box 1171 Blindern, 0318 Oslo, Norway; OSB@ous-hf.no; 4 Department of Oncology, Norwegian Radium Hospital, Oslo University Hospital, 0379 Oslo, Norway

**Keywords:** prostate-specific membrane antigen, prostate cancer, targeted alpha therapy

## Abstract

**Simple Summary:**

Currently, there is no treatment that can cure patients with late stage metastatic prostate cancer. Prostate-specific membrane antigen is a type of protein overexpressed on the membrane surface of most prostate cancer cells. The preclinical and clinical experiences in the rapidly evolving field of targeted alpha-particle radiation therapy for metastatic prostate cancer overexpressing prostate-specific membrane antigen are reviewed. Targeted alpha therapy employs radionuclides *emitting* highly energetic *alpha*-particles (cytotoxic payload) chelated to small molecules or monoclonal antibodies designed to target prostate-specific membrane antigen. In this review, we summarize the availability of therapeutic alpha-emitting radionuclides (terbium-149, astatine-211, bismuth-212 (lead-212), bismuth-213, radium-223, actinium-225, thorium-227), and the development of small molecules and antibodies targeting prostate-specific membrane antigen. The limitations in studies using animal models of human prostate cancer to accurately predict efficacy and toxicity in patients are addressed. We have attempted to also critically discuss hurdles related to logistical and supply aspects between different alpha-emitting prostate-specific membrane antigen-targeting radiopharmaceuticals. Lastly, we discuss the potentials, limitations, and future perspectives of prostate-specific membrane antigen-targeted alpha therapy.

**Abstract:**

Bone, lymph node, and visceral metastases are frequent in castrate-resistant prostate cancer patients. Since such patients have only a few months’ survival benefit from standard therapies, there is an urgent need for new personalized therapies. The prostate-specific membrane antigen (PSMA) is overexpressed in prostate cancer and is a molecular target for imaging diagnostics and targeted radionuclide therapy (theragnostics). PSMA-targeted α therapies (PSMA-TAT) may deliver potent and local radiation more selectively to cancer cells than PSMA-targeted β^−^ therapies. In this review, we summarize both the recent preclinical and clinical advances made in the development of PSMA-TAT, as well as the availability of therapeutic α-emitting radionuclides, the development of small molecules and antibodies targeting PSMA. Lastly, we discuss the potentials, limitations, and future perspectives of PSMA-TAT.

## 1. Introduction

Prostate cancer is the second most common cancer in men worldwide, with an estimated 1.3 million new cases and 359,000 deaths in 2018 [1]. The tumors of 10–20% of prostate cancer patients become refractory to androgen deprivation therapy and progress as metastatic castration-resistant prostate cancer (mCRPC) [2,3]. Bone metastases dominate, but lymph node and visceral metastases are also frequent in mCRPC patients [4,5,6]. Treatment options for mCRPC have expanded rapidly in the last 20 years [7,8,9,10,11]. The US Food and Drug Administration (FDA) and the European Medicines Agency (EMA) have introduced docetaxel (chemotherapy, approved in 2004/2007), cabazitaxel (chemotherapy, approved in 2010/2011), sipuleucel-T (autologous immunotherapy, approved in 2010/2013), abiraterone acetate (hormone therapy, approved in 2011/2011), enzalutamide (hormone therapy, approved in 2010/2013), Xofigo (radium-223, targeted alpha therapy, approved in 2013/2013), olaparib (PARP inhibitor therapy, approved in 2020/-), and rucaparib (PARP inhibitor therapy, approved in 2020/-) for the treatment of mCRPC [7,8,9,10,11]. However, mCRPC still remains incurable [11,12,13]. This may partly be explained by the inter-patient and intra-patient heterogeneity of the disease [14,15,16]. There is an urgent need for new personalized, highly effective targeted therapies for these patients. Several cell-surface proteins, such as glycoproteins, have been investigated as targets for the treatment of mCRPC [17,18,19]. The prostate-specific membrane antigen (PSMA), also known as glutamate carboxypeptidase II (GCPII) or folate hydrolase 1 (FOLH1), is one of the cell-surface proteins overexpressed in prostate cancer [20,21,22,23]. PSMA expression correlates with disease progression and Gleason score [20,21,22,24,25,26,27]. PSMA has a large extracellular domain, which can be recognized by antibodies, their fragments, small molecules, nanobodies, and aptamers [28,29]. Additionally, PSMA internalizes the bound targeting molecules and any payload attached to them, making it an excellent molecular target for both diagnostic imaging and targeted therapy, applying a theragnostic approach [24,30,31,32]. Many small molecules and antibodies targeting PSMA have been developed, labeled with β^−^ emitters (^177^Lu, ^161^Tb, ^131^I, ^90^Y, ^67^Cu, ^47^Sc), and studied in preclinical and clinical studies [33,34,35,36]. The small molecule radioligands ^177^Lu-PSMA-617 and ^177^Lu-PSMA-I&T are already being used in salvage/compassionate therapy in end-stage mCRPC patients [33,37,38]. A recent meta-analysis found a median progression-free survival of 11 months, overall survival of 13.7 months, and low toxicity profile [38]. Treatment of mCRPC patients with ^177^Lu-PSMA ligands give better therapeutic outcomes and cause fewer adverse effects than third-line treatment with enzalutamide and cabazitaxel [39]. Both radioligands are now being tested in phase 3 trials, VISION (^177^Lu-PSMA-617, NCT03511664) and SPLASH (^177^Lu-PSMA-I&T, NCT04647526). However, only 45% of mCRPC patients have a biochemical response (prostate-specific antigen (PSA) decline ≥ 50%) to ^177^Lu-PSMA-617, and around 30% of patients do not respond at all or are not suitable for this therapy due to diffuse red marrow infiltration [40,41,42]. Additionally, the presence of visceral metastases is associated with poor response and survival outcomes in mCRPC patients treated with ^177^Lu-PSMA [43]. Lu-177 emits β^−^ particles with a maximum energy of 497 keV and an average energy of 133 keV with a maximum soft-tissue penetration of 1.5 mm [44]. Such β^−^ particles will give a high absorbed dose to targeted macroscopic tumors while depositing a low absorbed dose to small cell clusters or single metastatic cancer cells because the range of the electrons is too long [45,46]. Preclinical and clinical studies suggest that radionuclide therapy with high linear energy transfer (LET) and short-range α emitters may have advantages over low LET β^−^ emitters [47]. PSMA-targeted α therapy (PSMA-TAT) may deliver potent and local radiation more selectively to cancer cells than PSMA-targeted β^−^ therapies. In this review, we summarize the recent advances made in preclinical and clinical development of PSMA-TAT, the availability of therapeutic α-emitting radionuclides, the development of small molecules and antibodies targeting PSMA, and discuss the potentials, limitations, and future perspectives of PSMA-TAT.

## 2. Prostate-Specific Membrane Antigen (PSMA)

**Figure 1:**
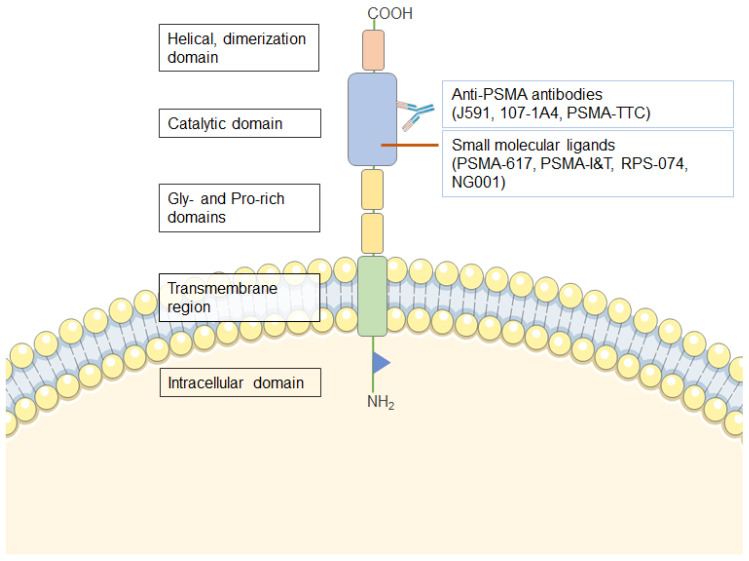
The simplified structure of prostate-specific membrane antigen (PSMA), its binding sites for PSMA ligands and antibodies (this figure is based on references [52,53,54]).

PSMA is a transmembrane glycoprotein of about 100 kDa with folate hydrolase, carboxypeptidase, and internalization activities [29]. It contains a transmembrane region of 24 amino acids, an N-terminal cytoplasmatic sequence of 19 amino acids, and a large extracellular domain of 707 amino acids (Figure 1). The extracellular domain of PSMA is highly glycosylated, and glycosylation is required for enzymatic activity [48,49]. Expression of PSMA is low in normal prostate tissue, kidneys, duodenum, salivary and lacrimal glands, brain, and intestines [50] but is increased in prostate cancer [21,23,32]. PSMA expression is correlated with prostate growth and significantly associated with the degree of differentiation and progression of mCRPC [29]. The precise mechanisms of the physiological function of PSMA in prostate cancer and its regulation are unknown. It has been suggested that elevated levels of PSMA enzymatic activity in prostate cancer cells increases cell folate uptake and proliferation and contributes to prostate carcinogenesis and progression [51].

## 3. Targeted Radionuclide Therapy of Prostate Cancer

Targeted radionuclide therapy (TRT) is a rapidly growing treatment modality for mCRPC [33,55,56]. TRT involves the use of radioisotopes or radiolabeled compounds that either naturally accumulate in or are designed to target and deliver a cytotoxic amount of radiation to prostate cancer cells sparing the surrounding normal tissues [55,56]. The systemic administration of tumor-targeted radiopharmaceuticals allows the simultaneous treatment of wide-spread bone and extraskeletal metastases [56,57], whereas a multiple and large-field irradiation using external beam radiotherapy is too toxic [58]. Currently, the β^−^-emitting radionuclide ^177^Lu is the most clinically used for TRT [37,38]. However, α particles present significantly higher energies than β^−^ particles, which combined with very short path lengths (<0.1 mm) result in high LET and a greater probability of generating DNA double-strand breaks upon interaction with cell nuclei (Table 1). This occurs almost independently of tissue oxygenation, dose rate, and cellular resistance to photon irradiation and chemotherapy [59]. Beta particle irradiation produces mainly single-strand breaks, exhibiting lower cytotoxic potency than α particles (Table 1). Consequently, the use of α emitters allows the specific targeting and killing of individual malignant cells while minimizing the toxicity to surrounding healthy tissue. Therefore, α particles are highly cytotoxic and promising candidates for TRT. 

### 3.1. Radionuclides Used for PSMA-TAT

Ra-223 (Xofigo) is the first-in-class and only α-emitting radiopharmaceutical currently approved by the FDA and the EMA for the treatment of mCRPC patients with symptomatic bone metastases and no known extraskeletal metastatic disease [63,64,65]. Ra-223 has a natural affinity for areas of high bone turnover because it is a bone-seeking calcium mimetic [66]. Targeted ^223^Ra therapy for mCRPC with its overall survival benefit has revolutionized the field of TRT [67,68]. Regional lymph node and visceral metastases, most frequently in lung and liver, are seen in approximately 8–15% of newly diagnosed patients but increase over time, currently affecting close to half of men during the course of their disease and represent the lethal phase of the disease [69,70]. Extraskeletal metastatic disease is now of growing concern in patients with mCRPC failing chemotherapy and/or the new oral anti-hormonal therapies targeting the androgen receptor axis (abiraterone and enzalutamide) evident by recent developments in diagnostic nuclear medicine; i.e., PSMA-positron emission tomography (PET) imaging [69,70]. An ideal TAT for mCRPC must exhibit a dual mode of action to combat the entire spectrum of metastases, both micrometastases and overt metastatic disease in lymph nodes, the skeleton, and visceral organs. The majority of bone, visceral, and lymph node metastases highly express PSMA [21,22]. Several α-emitting radionuclides, ^149^Tb, ^211^At, ^212^Pb/^212^Bi, ^223^Ra, ^225^Ac, and ^227^Th, and PSMA targeting agents are now being actively evaluated for PSMA-TAT (Table 2, Table 3, Table 4 and Table 5). The availability, physical and chemical properties of radionuclides play an important role in identifying α-emitting radionuclides suitable for therapy. The half-life of the radionuclide should neither be too long to avoid extended radiation effects after radiopharmaceutical administration nor too short to have enough time for logistical aspects, such as production and transportation.

**Figure 2:**
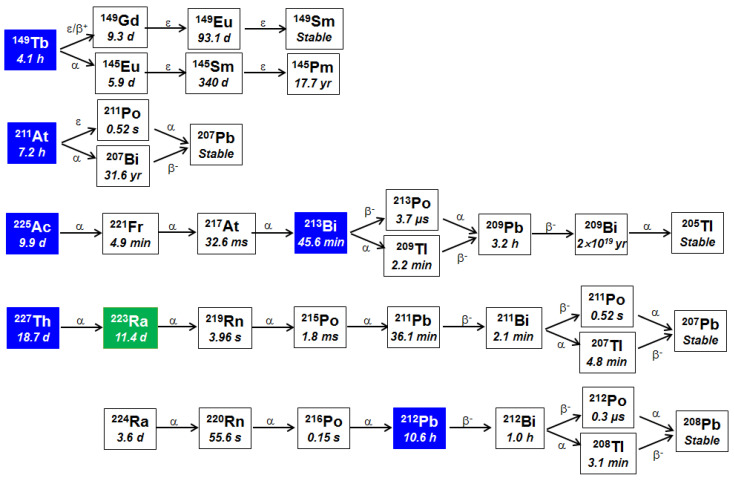
Simplified decay chains of ^149^Tb, ^211^At, ^225^Ac, ^227^Th, ^223^Ra, and ^224^Ra radionuclides. α–alpha particle, β–beta particle, β^−^–electron, β^+^–positron, ε–electron capture. Information extracted from the National Nuclear Data Center database, Brookhaven National Laboratory [75].

The radiopharmaceuticals must be sufficiently stable compounds both in vitro and in vivo. The nuclear recoil energy of about 100–200 keV from α-decay is sufficient to break chemical bonds between the targeting moiety and the radionuclide, which can lead to circulating carrier-free daughter radionuclides in blood [114,118,119,120,121]. The decay of the parent radionuclides ^212^Pb/^212^Bi, ^225^Ac, ^223^Ra, and ^227^Th form multiple α-emitting daughter radionuclides (Figure 2). The released daughter radionuclide can be retained inside the tumor and enhance the cytotoxic effect, but if the released daughter radionuclide has a long enough half-life, it can redistribute within the body and damage healthy tissues [118,120,122].

### 3.2. PSMA Targeting Agents in TRT

The extracellular domain makes up to 95% of PSMA and provides an accessible target for small molecules, antibodies, and their fragments [29,80]. A number of PSMA-targeting small molecules (also denoted as ligands or inhibitors) and antibodies have been developed for molecular imaging by single-photon emission computed tomography (SPECT) and PET and/or TRT [29,30,123,124]. Small molecules offer potential advantages over antibodies, such as small size, easy synthesis and modification, high specificity and affinity, good permeability with rapid accumulation in tumor, and rapid clearance from most normal tissues (Table 3).

Many different PSMA ligands have been developed [124,125,126,127], and several of them have been investigated for TAT in preclinical and clinical studies (Table 4 and Table 5). Small molecule PSMA ligands consist of three components: a binding entity (motif), a linker (spacer), and a radiolabel-bearing moiety (a bifunctional chelator for radiolabeling with metal radionuclides or a prosthetic group for astatination) [41,125,126,128]. The linker region is used to adjust the molecular size and polarity to impact the in vivo distribution properties [125,126,127,129,130,131,132,133]. The first small molecule inhibitors targeting PSMA, based on the glutamate-urea-lysine entity (MIP-1072 and MIP-1095), were introduced into the clinic in 2013 for prostate cancer imaging with ^123^I [134]. These inhibitors showed a rapid advantageous localization in tumor lesions, including soft tissue and bone metastases. In 2014, German research groups from Heidelberg and Munich developed PSMA-11 (also called PSMA-HBED-CC) and PSMA I&T ligands, respectively [135,136]. In 2015, the Heidelberg group developed PSMA-617 [130]. In 2020, the FDA approved ^68^Ga-PSMA-11 as the first drug for PET imaging of PSMA positive lesions in men with prostate cancer [137]. Unfortunately, the chelator HBED-CC (N,N’-bis [2-hydroxy-5-(carboxyethyl)benzyl] ethylenediamine-N,N’-diacetic acid) used in PSMA-11 is not suitable for the stable complexation with therapeutic radionuclides [138]. To date, PSMA-617 and PSMA I&T are the most commonly used theragnostic PSMA radioligands because they can be radiolabeled with diagnostic, e.g., ^68^Ga and ^44^Sc for PET and ^111^In for SPECT, as well as therapeutic radiometals [138,139,140,141,142,143,144]. In an attempt to prolong circulation in the blood and therewith, to increase the dose delivered to tumors and the tumor-to-kidney ratio, PSMA ligands have been structurally modified by adding albumin-binding moieties [123,132,145,146,147,148,149,150]. Preclinical and clinical studies are required to demonstrate whether PSMA inhibitors with enhanced albumin binding (HTK01169, PSMA-ALB-56, RPS-074, EB-PSMA-617, CTT1403) can increase the efficacy of PSMA-TRT [103,146,148,150,151]. A recently published review provides a comprehensive overview of the current status of selected PSMA inhibitors that have been developed from 1996–2020, emphasizing recent synthetic advances and chemical strategies while highlighting the therapeutic potential and drawbacks of each inhibitor [152].

Several antibodies and their fragments are also being evaluated for TRT in preclinical and clinical studies [97,104,153,154]. The antibodies J591, 107-1A4, PSMA-TTC, and their conjugates recognize extracellular epitopes of PSMA (Figure 1) and are the most investigated for TAT [81,96,97,104,154]. J591, 107-1A4, and PSMA-TTC were described and characterized in 1997 by Liu et al. [155], in 1998 by Brown et al. [156], and in 2020 by Hammer et al. [104], respectively. The half-life of the radionuclide must be matched with the antibody residence time in the tumor to deliver the maximum irradiation dose [157]. Antibodies have a plasma half-life of about 2–7 days, and several days are needed to reach maximum tumor uptake (Table 3). Radionuclides with too short or too long half-lives may lead to suboptimal efficacy or toxicity.

### 3.3. Radium-223 for PSMA-TAT

Among all α-emitting radionuclides, ^223^Ra has the most suitable half-life (t_1/2_ = 11.4 days), decay properties, and safety based on extensive clinical use as Xofigo for TAT. Ra-223 decays via a chain of five short-lived daughter radionuclides (t_1/2_ from 0.5 s up to 36.1 min) to stable ^207^Pb, emitting four α particles and two β^−^ particles (Figure 2). No redistribution of radioactive daughters has yet been reported [66,158]. Consequently, high decay energy (27–28 MeV) is released over a short range [66,158,159]. Ra-223 can be produced in large quantities from ^227^Ac (t_1/2_ = 21.7 years) generator [66]. The half-life of ^223^Ra provides sufficient time for its preparation, distribution, and administration to patients. Unfortunately, due to the bone-seeking characteristics of *^223^*Ra, its clinical use is limited to prostate cancer patients with osteoblastic metastases [66]. Chelate complex formation is essential to treat extraskeletal cancer metastases. A number of bifunctional chelators, which carry a functional unit for the immobilization of the radiometal and a functional group for the covalent attachment to a biological target molecule, have been tested to chelate ^223^Ra [160,161]. Unfortunately, ^223^Ra, like other alkaline earth metals, forms very weak complexes [160,161]. To overcome this limitation, several nanomaterials, hydroxyapatites, polyoxopalladates, nanozeolites, barium sulfate, and titanium dioxide nanoparticles have been tested for stable incorporation of radium and linking to the targeting vector [84,162,163,164,165,166]. So far, only one paper has been published investigating the encapsulation of ^223^Ra into functionalized nanozeolites for PSMA-TAT [84]. NaA zeolite is one of the synthetic, non-toxic microporous crystalline aluminosilicate zeolites that can accommodate a wide variety of various molecules, including radionuclides [165,167]. Czerwińska et al. [84] modified NaA nanozeolite with silane-PEG (polyethylene glycol) and functionalized it with anti-PSMA D2B antibody as a carrier of ^223^Ra and its daughter radionuclides for PSMA-TAT. The obtained 120 nm ^223^RaA–silane-PEG-PSMA D2B bioconjugate was highly stable (<2% of ^223^Ra and <6% of the daughter radionuclides were released up to 12 days) [84]. The radioimmunoconjugate bound specifically and internalized into PSMA-expressing C4-2 cells, but not into PSMA-negative DU-145 cells. Treatment of C4-2 cells with 20 kBq/mL of the radioimmunoconjugate resulted in an 80% reduction in metabolic activity [84]. Further preclinical studies in vivo are needed to validate the therapeutic efficacy and toxicity of ^223^RaA-silane-PEG-D2B. It is too early to know if this approach will be successful.

### 3.4. Bismuth-213 for PSMA-TAT

Bi-213 is a mixed α and β^−^ emitter with a half-life of 45.6 min (Figure 2). Preclinical studies have demonstrated that TAT with ^213^Bi labeled antibody J591, small molecule inhibitor PSMA-I&T or nanobody JVZ-008 showed efficient and rapid tumor targeting, induced apoptosis in PSMA-overexpressing cell lines, significantly delayed spheroid growth of xenograft tumor growth in nude mice [81,96,97,98]. Small molecule PSMA-I&T induced more double-strand breaks than nanobody JVZ-008 [98]. A dosimetry estimate comparing ^213^Bi-PSMA-617 and ^225^Ac-PSMA-617 has demonstrated that the short-lived ^213^Bi is an inferior choice for TAT with PSMA-617 [168]. To our knowledge, there is only one single case report to date on the clinical application of ^213^Bi-PSMA-617 [105]. The patient was treated with two cycles of ^213^Bi-PSMA-617 with a cumulative activity of 592 MBq. A biochemical response (decrease in PSA level from 237 μg/L to 43 μg/L) was observed [105]. The short half-life of ^213^Bi makes this radionuclide less suitable for routine therapeutic applications due to logistical challenges. Hence, the subject is not discussed further.

### 3.5. Astatine-211 for PSMA-TAT

At-211 has a half-life of 7.2 hours (Figure 2) and is a promising radiohalogen for PSMA-TAT [86,87,88]. It decays by electron capture (58.3%) to ^211^Po (t_1/2_ = 0.52 s), which decays to stable ^207^Pb by emitting an α particle (7.45 MeV, 100%). Additionally, its daughter ^211^Po emits K X-rays in the range of 77 to 92 keV that allows the quantification of ^211^At radioactivity and scintigraphic imaging of ^211^At in vivo [169]. In 2009, modified anti-PSMA antibody 107-1A4 was labeled with ^211^At [86]. SCID mice bearing intra-tibial human prostate cancer C4-2B tumors were treated with 370 kBq ^211^At-107-1A4 [86]. The treatment decreased PSA levels in mice without any toxicity. The same year urea-based PSMA inhibitor labeled with ^211^At, 2-[3-[5-(4-[^211^At]astato-benzoylamino)-1-carboxy-pentyl]-ureido]-pentanedioic acid (ABCPUP) was synthesized [82]. In vitro studies demonstrated that ^211^At-ABCUP had significantly higher uptake in PC3 PIP (PSMA-positive) compared to PC3 (PSMA-negative) human prostate cancer cells. In 2016, Kiess et al. [88] synthesized a urea-based small-molecule targeting PSMA, (2*S*)-2-(3-(1-carboxy-5-(4-^211^At-astatobenzamido) pentyl)ureido)-pentanedioic acid (^211^At-PSMA 6), that significantly improved survival in mice bearing PC3 PIP micrometastases. However, the high uptake of ^211^At-PSMA 6 in renal proximal tubules resulted in late nephrotoxicity (≤12 months) [88]. The maximum tolerated single dose of ^211^At-PSMA 6 in immunocompetent CD1 mice was 37 kBq, and the lethal dose to 10% of mice was 111 kBq [88]. Such toxicity limits the clinical use of ^211^At-PSMA 6. In 2017, Kelly et al. [89] designed six novel urea-based ligands for dual-targeting PSMA and human serum albumin. Compounds with higher affinity for human serum albumin showed prolonged blood retention resulting in reduced kidney uptake. Their most potent compounds, RPS-027 and MIP-1095, were labeled with ^131^I as a surrogate for ^211^At, and their biodistribution was tested in mice bearing LNCaP xenograft tumors [89]. Similar tumor uptake was observed for both products, but ^131^I-RPS-027 had a five-fold reduction in kidney uptake compared to MIP-1095. Recently, to reduce kidney uptake, protein-Glu-urea-Lys conjugates and potential metabolites were synthesized and radio iodinated (as the surrogate for ^211^At) and administered to athymic mice bearing C4-2B tumor xenografts [90]. Conjugation of PEGylated PSMA derivatives to proteins reduce kidney uptake, but long polyethylene glycol (PEG) linkers have reduced uptake in tumor [90]. There are not many studies testing ^211^At for PSMA-TAT since the availability of ^211^At is limited. At-211 is produced mostly in cyclotrons by the bombardment of natural ^209^Bi with α-beam at energies below 28.4 MeV [169,170]. Unfortunately, there are only about 30 cyclotrons in the world that have the beam characteristics required for ^211^At production [169].

### 3.6. Actinium-225 for PSMA-TAT

Ac-225 (t_1/2_ = 10 days) decays via a chain of five daughter radionuclides (t_1/2_ from 3.7 µs up to 45.6 min) to stable ^209^Bi, emitting four α particles and two β^−^ particles (Figure 2). Earlier, the potential use of ^225^Ac for TAT was limited because of difficulties in the selection of chelating agents able to form strong bonds, leading to renal toxicity induced by the longer-lived decay product ^213^Bi [55,121]. Nevertheless, some stable complexes have been synthesized, and several more recent clinical trials have demonstrated the potential of ^225^Ac-PSMA-617 and ^225^Ac-PSMA-I&T for the treatment of mCRPC (Table 5). The first-in-human PSMA-TAT study was published in 2016 by Kratochwil et al. [42] from Heidelberg. Two mCRPC patients with challenging clinical situations and extensive pretreatment were treated with 100 kBq/kg of ^225^Ac-PSMA-617 at bi-monthly intervals as salvage therapy after the presence of a PSMA-positive tumor phenotype had been validated by ^68^Ga-PSMA-11 PET/CT [42]. The first patient was not suitable for ^177^Lu-PSMA-617 (diffuse red marrow infiltration), and the second one was resistant to ^177^Lu-PSMA-617 [42]. Both patients showed a complete response on the PET/CT scan, and PSA declined below the measurable level [42]. Salivary gland toxicity leading to dry mouth syndrome or xerostomia was reported in both patients [42]. The second study with 14 mCRPC patients found that a treatment activity of 100 kBq/kg of body weight of ^225^Ac-PSMA-617 per cycle every 8 weeks was the most optimal when considering both efficacy (biochemical response) and tolerability [106]. Severe xerostomia was the dose-limiting toxicity [106]. This standardized treatment protocol for ^225^Ac-PSMA-617 is routinely applied for salvage therapy of end-stage mCRPC patients in many studies [106,107,113]. The efficacy of ^225^Ac-PSMA-617 TAT was evaluated in 40 mCRPC patients [107]. This study demonstrated a PSA decline of more than 50% in 63% of patients, with a median duration of tumor control of 9 months [107]. The median overall survival was more than 12 months [107]. The majority of mCRPC patients at the Heidelberg clinic were heavily pretreated before TAT with chemotherapy, radiotherapy, and androgen deprivation therapy (abiraterone (85%) and enzalutamide (60%)) [107]. In South Africa, chemotherapy-naïve mCRPC patients treated with ^225^Ac-PSMA-617 TAT had reduced toxicity to salivary glands, a 90% PSA decline in 88% of the patients, and 50% achieved undetectable serum PSA and remained in remission 12 months after therapy [109]. The first clinical data using ^225^Ac-PSMA-I&T showed highly comparable biochemical responses as after ^225^Ac-PSMA-617 TAT [116,117].

Since PSMA-617 crosses the blood–brain barrier and accumulates in cerebral metastases [171], a significant regression of cerebral metastases was demonstrated using *^225^*Ac-PSMA-617 [108]. Prostate cancer patients with brain metastases have limited treatment options and poor survival, and TAT with *^225^*Ac-PSMA-617 may have substantial therapeutic potential for these patients.

In the clinical setting, several studies reported toxicity related to TAT with ^225^Ac-PSMA-617/PSMA-I&T (Table 5). Xerostomia is a common side effect that causes 10–25% of patients to stop TAT with ^225^Ac-PSMA [42,107,111,112,117,172]. Xerostomia should, therefore, be prevented. Modification of the administered activity of ^225^Ac-PSMA-617 and the number of cycles of TAT may decrease the side effects while still achieving response [111,173]. Sialendoscopy with dilatation, saline irrigation, and steroid injection (prednisolone) have been investigated in patients with some but limited success [174]. A case report in one patient describes the potential beneficial effects of intraparenchymal injections of botulinum toxin before ^225^Ac-PSMA-617 TAT [175]. External cooling of the salivary gland using ice packs from 30 min pre-infusion through 2 h post-infusion of radiopharmaceuticals was expected to reduce PSMA radioligand uptake due to vasoconstriction [172]. However, the relative contributions of salivary gland cooling and the reduced ^225^Ac-PSMA-617 activity in minimizing xerostomia severity remain unclear. Therefore, effective methods to reduce salivary toxicity are needed.

Due to the physiological expression of PSMA in kidneys and predominantly renal excretion of ^225^Ac-PSMA-617, there is concern about possible radiation toxicity to the kidneys that may cause acute and long-term effects [138,176,177]. It has been reported that the kidney function deteriorated in a patient with one functional kidney after ^225^Ac-PSMA-617 [109] and that chronic kidney disease was found in two patients with mCRPC after ^225^Ac-PSMA-617 therapy [114]. Until now, retention times of PSMA ligands either in kidneys or in tumor cells have not yet been evaluated systematically [168]. If PSMA on the surface of cancer cells is not sufficiently internalized after binding of the ligand, TAT with ^225^Ac with multiple unstable daughters might be suboptimal and toxic [168]. It has also been speculated that the radioactive daughters of ^225^Ac, but not ^225^Ac-PSMA-617, can accumulate in the tubular cells and irradiate the kidneys, leading to renal injury [114]. In such cases, the therapeutic potential of ^225^Ac-PSMA will be substantially decreased, and toxicity increased. Further studies are necessary to evaluate the stability, retention times, and intracellular localization in cancer cells and kidneys of the ^225^Ac-PSMA complexes intended for TAT.

The first report on the use of ^225^Ac-PSMA-617 in vivo was only published in 2019 [99]. NSG mice bearing subcutaneous C4-2 tumors were treated with 20, 40, and 100 kBq/mouse of ^225^Ac-PSMA-617 [99]. Significant tumor growth inhibition was observed in all treatment groups compared to the control. However, mice treated with 100 kBq had some weight loss, while the mice treated with lower activities experienced only transient weight loss. In 2020, the same group reported a useful mouse model of human metastatic prostate cancer by injecting C4-2 cells expressing firefly luciferase into the left ventricle of NSG mice, which was then used to evaluate the effectiveness of ^225^Ac-PSMA-617 at various disease stages [100]. Early treatment, one-week post-inoculation of C4-2 cells, with 40 kBq/mouse of ^225^Ac-PSMA-617 prevented liver metastases and led to significant survival benefit [100]. In 2019, Kelly et al. [103] studied the albumin-binding and PSMA-targeting ligand RPS-074 labeled with ^225^Ac in BALB/c mice bearing LNCaP xenograft tumors. Significant tumor growth inhibition was observed in mice treated with 74 and 148 kBq of ^225^Ac-RPS-074. A single injection of 148 kBq induced a complete response in 6 of 7 tumors, with no apparent toxic effects. In 2020, Current et al. [101] documented that ^225^Ac-PSMA-617 efficacy is dependent on cellular PSMA levels and intra-tumoral PSMA heterogeneity.

Another approach to reducing toxicity in salivary glands and kidneys is to use antibodies (e.g., J591) instead of small molecule PSMA for TAT [83,154]. J591 binds to a different site of PSMA compared to PSMA ligands and has a much lower distribution in salivary glands and kidneys [154]. Ongoing clinical trials with ^227^Ac-J591 will provide the missing information on stability, efficacy, and toxicity (Table 6).

The mainly retrospective studies have reported promising response rates, progression-free survival, and overall survival (Table 5). The limited availability of ^225^Ac is the main challenge for its clinical use. Many research centers investigate the possibility of producing ^225^Ac in commercial quantities. In the future, scaled-up production of ^225^Ac could be achieved by the use of a high current cyclotron or electron linear accelerator (linac) [178,179].

### 3.7. Thorium-227 for PSMA-TAT

Th-227 has a physical half-life of 18.7 days and decays through radioactive ^223^Ra and the other short-lived radionuclides in its decay chain to stable ^207^Pb by emitting five α particles (Figure 2). The long half-life of ^227^Th allows transportation and preparation of the radiopharmaceutical. Due to its chemical properties, ^227^Th can be linked to a variety of antibodies and proteins [72,180]. Th-227 complexed with octadentate 3,2-hydroxypyridinone (3,2-HOPO) chelators that are conjugated to antibodies or other targeting moieties results in highly stable targeted ^227^Th conjugates (TTCs) [104]. TTCs, therefore, represent a new promising class of TAT [104]. A novel, fully human antibody-based TAT, PSMA-TTC (BAY 2315497), has been recently developed by Bayer and tested in mice bearing prostate cancer tumors [104]. The antitumor efficacy of PSMA-TTC was observed in different prostate cancer models. However, survival and long-term toxicity have not been reported. These preclinical data encouraged the further investigation of BAY 2315497 in an ongoing phase I trial in mCRPC (Table 6).

### 3.8. Terbium-149 for PSMA-TAT

A very interesting alternative to the presented α emitters is the ^149^Tb, currently studied in preclinical radioimmunotherapy [85]. Tb-149 has a half-life of 4.1 hours and is considered a promising theragnostic radionuclide. It decays by α emission (3.97 MeV, 16.7%), as well as electron capture (76.2%), positron emission (7.1%), gamma rays, and X-rays. Therefore, it can be used for TAT and PET imaging [181]. Longer half-life of ^149^Tb compared to ^213^Bi (t_1/2_ = 46 min) and ^212^Bi (t_1/2_ = 1.0 h), absence of α-emitting daughters, and chelation with S-2-(4-isothiocyanatobenzyl)-1,4,7,10-tetraazacyclododecane-1,4,7,10-tetraacetic acid (DOTA) are clear advantages of ^149^Tb [181]. Antitumor activity of ^149^Tb-PSMA-617 has been demonstrated in mice bearing PC3 PIP tumors [85]. Tb-149 is a rare-earth element, and its production and chemical separation are associated with serious difficulties, which partially explains why ^149^Tb is not in clinical use yet [182]. Additionally, the presence of long-lived daughters appearing during the decay of ^149^Tb (Figure 2) complicates dosimetry and might increase the risk of undesirable physiological effects. The long-lived daughter radionuclides, ^149^Eu (t_1/2_ = 93 d) and ^145^Sm (t_1/2_ = 340 d), are potential bone seekers [183]. Due to the recoil energy of the alpha decay of ^149^Tb, there is a high risk of release of these radionuclides into the bloodstream leading to potential accumulation in bone and, thus, to long-term irradiation of the bone marrow [183].

### 3.9. Lead-212 for PSMA-TAT

Another suitable radionuclide for PSMA-TAT is β^−^ emitter ^212^Pb (t_1/2_ = 10.6 h) that acts as an in vivo generator of α particles via its short-lived progenies ^212^Bi (t_1/2_ = 60.6 min) and ^212^Po (t_1/2_ = 0.3 µs) (Figure 2). The decay of one ^212^Pb atom releases on average one α particle and a mean α energy of 7.9 MeV. The use of ^212^Pb circumvents the impractical short half-life of ^212^Bi and delivers around ten times the dose per administered activity unit compared to ^212^Bi alone, reducing the amount of activity required [184,185]. Pb-212 can be obtained at an industrial scale from ^224^Ra-based generators using ^228^Th (t_1/2_ = 1.9 years) as a long-term generator. It has suitable properties in terms of chelation chemistry and forms stable complexes both with the versatile chelator DOTA and the lead-specific chelator S-2-(4-isothiocyanatobenzyl)-1,4,7,10-tetraaza-1,4,7,10-tetra(2-carbamoylmethyl)-cyclododecane (TCMC) [186]. Comprehensive reviews describing the use of ^212^Pb for TAT of cancer have recently been published [170,187,188].

Several research groups have developed PSMA-targeting ligands specifically designed for ^212^Pb chelation that show promising tumor-targeting in preclinical models [91,92,94,95]. The urea-based PSMA ligands NG001, labeled with ^212^Pb, and CA012 and L2, labeled with the surrogate ^203^Pb, show high tumor uptake (8–25%ID/g) 1–2 h post-injection in mice bearing PSMA-positive tumors, which is comparable with clinically used ^177^Lu-PSMA-617 [91,92,94,130,148]. In NSG mice bearing PC3 PIP tumors, 1.5 and 3.7 MBq of ^212^Pb-L2 significantly inhibited tumor growth with therapeutic indices (TI) of 1.9 and 3.0, respectively, with time to reach a 10-fold tumor increase used as an endpoint [92]. In a micrometastatic tumor model, 3.7 MBq of ^212^Pb-L2 demonstrated an increased survival benefit compared to 37 MBq of ^177^Lu-PSMA-617 (TI of 1.2 vs. 1.0, respectively). However, a long-term toxicity study of ^212^Pb-L2 in healthy, immunocompetent mice identified the kidney as the dose-limiting organ, and the maximum tolerated dose (MTD) of the radioligand was determined to be 1.5 MBq. In athymic nude mice bearing C4-2 xenografts, an injected activity of only 0.32 MBq of ^212^Pb-NG001 demonstrated increased survival compared to the control group with a therapeutic index of >2.0 (median survival of 15 days vs. >30 days) [93]. The results warrant further preclinical studies to evaluate the long-term toxicity of ^212^Pb-NG001.

With shorter-lived radionuclides, such as ^212^Pb, the high initial kidney uptake could present a potential toxicity problem because of the higher dose rate of these radionuclides [189]. However, the mentioned Pb-labeled ligand NG001 exhibit lower kidney uptake than PSMA-617 [94]. Promising therapeutic results labeled with the short-lived ^213^Bi (t_1/2_ = 45.6 min) with an even higher dose rate than ^212^Pb were reported [105]. Another challenge with ^212^Pb is the retention of daughter radionuclides in the chelator after decay. Up to 36% of ^212^Bi could dissociate from DOTA and TCMC chelators of antibody complexes because of high recoil energies of the α-emitting daughters [120,184,190,191]. However, no translocation of the ^212^Bi daughter was detected in non-targeted organs during a 24 hour study period of ^212^Pb-NG001, likely prevented by the rapid tumor targeting and cellular internalization, as well as the rapid normal tissue clearance of the radioligand [94].

### 3.10. Dual Alpha (^224^Ra&^212^Pb) for TAT

An ideal PSMA-TAT for mCRPC must combat the entire spectrum of metastases present in the patients. A dual-alpha approach that uses the high LET ionizing energy of ^224^Ra and daughter radionuclides to target various mCRPC lesions have been presented [95]. Here, a ^224^Ra solution in transient equilibrium with daughter radionuclides was used for in situ labeling of a PSMA-targeting ligand, i.e., ^212^Pb is complexed by the ligand in the presence of ^224^Ra [95,184,185]. The resulting solution has dual-targeting properties; natural bone-seeking ^224^Ra will target osteoblastic metastatic lesions, and the ^212^Pb-labeled PSMA ligand will target extraskeletal metastases by selective binding to the surface of PSMA-expressing cells [95]. Thus, the dual-alpha approach is providing a bone-seeker and a PSMA-seeker in one radiopharmaceutical solution. The accumulation of ^224^Ra in bone and ^212^Pb-labeled PSMA ligand in tumor sites was verified in C4-2 tumor-bearing mice and warrants further investigation in vivo [95].

### 3.11. Combination Treatments with PSMA-TAT

Around 20% of patients treated with ^225^Ac-PSMA-617 have a poor response (Table 5) or early resistance against ^225^Ac-PSMA-617 [107], despite sufficient expression of PSMA and uptake of ^225^Ac-PSMA-617 in their tumors [192]. Several combination treatments with PSMA-TAT have been proposed [173,192,193]. Their goal is to increase PSMA-TAT efficacy by using therapies with different action mechanisms together with TAT, keeping toxic effects to a minimum. The tandem PSMA-RLT approach has been introduced to increase efficacy and reduce toxicity [173,194]. A single course of tandem therapy with low-activity ^225^Ac-PSMA-617 and full-activity ^177^Lu-PSMA-617 has enhanced response to PSMA-RLT and minimized xerostomia severity in men with late-stage/end-stage mCRPC [173,194]. There is a subgroup of patients (~17%) with poor response to ^225^Ac-PSMA-617 who harbor mutations in DNA damage–repair and checkpoint genes [192]. Combining PSMA-TAT and DNA damage–repair–targeting agents, such as poly(ADP-ribose)-polymerase inhibitors, have been suggested for these patients [192]. Lastly, it has been hypothesized that PSMA-TAT may increase tumor immunogenicity, and the use of immune checkpoint inhibitors may improve efficacy [102]. Synergistic antitumor efficacy between ^225^Ac-PSMA-617 and PD-1 blockage has recently been observed in C57BL/6-mice bearing syngeneic RM1-PGLS tumors [102].

## 4. Limitations of Preclinical Studies Related to Clinical PSMA-TAT

A major concern of PSMA-targeting radioligands is the radiotoxicity in PSMA-expressing organs. Only the most optimal and safest PSMA-targeted radioligands from preclinical studies are tested in humans. However, radiotoxicity in rodents is not the most accurate predictor of toxicity in humans because the human, rat, and mouse PSMA have different patterns of anatomical expression in normal tissues [195,196,197]. For example, PSMA levels in human submandibular gland are approximately four-fold higher compared with mouse and fifty-four-fold higher compared with rat submandibular gland [197]. Rupp et al. demonstrated that the accumulation of PSMA ligands in salivary glands in humans is high due to PSMA-unrelated uptake mechanisms [198], while PSMA radioligands (^177^Lu, ^212^Pb) show negligible uptake in mouse salivary glands [94,95,132]. The details of salivary glands’ uptake mechanisms are currently unclear and must be further investigated using different clinical models. Furthermore, PSMA levels in human kidneys are approximately two-fold lower compared with mouse kidneys [138]. Many patients treated with PSMA-TAT report severe xerostomia, and only a few of them, nephrotoxicity (Table 5). The direct comparison of PSMA-TAT data from various groups is not easy since different mouse strains, cell lines, and different specific activities of radiopharmaceuticals have been used in the various studies. PSMA-TAT efficacy increases with increasing PSMA levels [101]. For example, transduced PC3 PIP cells present much higher PSMA expression levels than LNCaP or C4-2 cells (Table 7), and the direct comparisons of survival, tumor uptake, and tumor to kidney ratio of different radiopharmaceuticals must be done carefully.

To date, PSMA-617 and PSMA-I&T are the most often clinically applied small molecule ligands for TRT and diagnostics [39,202]. Both ligands contain a peptidomimetic glutamate–urea–lysine binding motif, but chelators are different [138,203]. PSMA-617 contains a DOTA chelator while PSMA I&T contains a DOTAGA (1,4,7,10-tetraazacyclododecane-1-(glutamic acid)-4,7,10-triacetic acid) chelator, [138,203]. Biodistribution studies of ^177^Lu-PSMA-617 and ^177^Lu-PSMA-I&T in mice bearing PC3 PIP or PC295 PDX prostate tumors demonstrated comparable tumor uptake at all time points, while ^177^Lu-PSMA-I&T had much higher (10–40 folds) renal uptake, resulting in an unfavorable tumor-to-kidney ratio [204,205]. The data from these preclinical studies are in partial discrepancy with clinical studies: biodistribution and absorbed doses in tumor or kidneys were comparable for PSMA-617 and PSMA-I&T [206]. Therefore, caution must be taken, so that novel PSMA ligands with clinical potential are not discarded based on preclinical data.

## 5. Future of PSMA-TAT

The success of PSMA-TAT depends on the availability of α emitters and chelators, enhanced tumor uptake of linkers and targeting moieties, and reduced toxicity and progeny redistribution.

Further preclinical proof of concepts with PSMA-TAT in relevant murine models of mCRPC regarding the impact of dose rate and relative biological effectiveness is sorely needed. This also relates to direct comparisons of ^225^Ac, ^227^Th, and ^212^Pb PSMA-TAT with ^177^Lu-PSMA TRT.

The detailed mechanisms of action of PSMA-TAT are far from fully understood [72]. Elucidating in vitro and in vivo molecular and cellular mechanisms related to targeted and bystander effects of PSMA-TAT are important. It has been suggested that TAT bears the potential to induce immunogenic cell death through the release of damage-associated molecular patterns from dying tumor cells that result in the activation of tumor-specific immune responses [207]. However, the role of PSMA-TAT in modulating the immunogenicity of prostate cancer cells remains unknown.

The dosimetry for the labeled α emitter and its progeny is challenging and still in the early stage because of the heterogeneous antigen expression among cancer cells and the nature of short-range, high-LET alpha radiation (Table 1 and Table 2). This needs to be further investigated using modeling methods. Autoradiographs can help to obtain high-resolution images, but they can only be performed ex vivo. In such cases, estimates of radiation doses could be based on experimental data and modeling. Recommended therapeutic activities (e.g., 4–8 MBq ^225^Ac-PSMA-617) limit the clinical applicability of SPECT [208]. Theranostic imaging protocols using ^68^Ga-PSMA ligands does not provide any information about the translocation of daughter radionuclides, and the impact of recoiling daughter molecules on dosimetry of α-emitting radiopharmaceuticals on healthy tissues are unknown.

## 6. Conclusions

Preclinical studies and preliminary efficacy and safety data on PSMA-TAT in mCRPC patients are very encouraging. Because of production cost, logistical, and supply problems with several of the more promising α-emitters, the future of clinical PSMA-TAT should focus on radionuclides that are suitable for large scale production and supply, such as ^212^Pb, which can be produced at the industrial scale with existing methods. Dual targeting with ^212^Pb/^224^Ra is particularly promising since most late-stage prostate cancers have skeletal involvement, and ^212^Pb-PSMA ligand and ^224^Ra represent two different targeting approaches in one radiopharmaceutical solution. The future of clinical PSMA-TAT may also include novel PSMA ligands and combined approaches.

## Figures and Tables

**Table 1:** Comparison of conventional external radiation radiotherapy and targeted radionuclide therapy [60,61,62]. AMU, atomic mass units LET, linear energy transfer; DBS, double-strand breaks; SSB, single-strand breaks.

Properties	Targeted Radionuclide Therapy		External Radiation Therapy
	α Emitters	β^−^ Emitters	χ- and γ-rays
Type	Helium nucleus	Electron	Photon
Mass (AMU)	4 (heavy)	0.0005 (light)	0 (massless)
Speed of light	6% (slow)	98% (fast)	100% (fast)
Initial energy	4–9 MeV	0.05–2.3 MeV	0.035–3 MeV
Range in tissue	0.04–0.1 mm	0.05–12 mm	centimetres
LET	50–230 keV/µm	0.1–1.0 keV/µm	0.2 keV/µm
DNA damage	Irrepairable (DSB)	Repairable (SSB)	Repairable (SSB)
Ionizing ability	Very high	Medium	Low
Number of DNA hits to kill cells	1–5	100–2000	>>1000
Irradiation field	Whole-body	Whole-body	Region
Dose distribution	Very heterogeneous	Heterogeneous	Homogenous
Dose rate	<1 Gy/h	<1 Gy/h	60–120 Gy/h, in 2 Gy fractions
Effect of oxygen on cell killing	Weak	Moderate	Strong
Bystander effect	Yes	Yes	Yes
Crossfire radiation	Yes	Yes	Yes

**Table 2:** Overview of α-emitting radionuclides used in PSMA-TAT [71,72,73,74,75].

Radio- Nuclide	Half- Life	Main Production Method	Radio- Nuclide Availability	Emitted Particles	Total α Energy Emitted per Decay (MeV)	Range in Tissue (µm)	LET (keV/µm)	Emissions for Imaging
^149^Tb	4.1 h	Accelerator	Moderate	1α, 1ε/2ε, 1β^+^/2β^+^	0.7 *	25	140	165 and 730 keV
^211^At	7.2 h	Cyclotron	Moderate	1α, 1ε	6.9	55–80	71–230	77–92 keV
^212^Pb/ ^212^Bi	10.6 h	^224^Ra generator	Very high	1α, 2β^−^	7.9	40–100	61–230	238 keV
^213^Bi	45.6 min	^225^Ac generator	Moderate	2α, 2β^−^	8.5	40–100	65–230	440 and 1566 keV
^223^Ra	11.4 days	^227^Th generator	High	4α, 2β^−^	26.8	46–70	71–230	84, 95, 144, 154 and 270 keV
^225^Ac	9.9 days	^229^Th generator; cyclotron, accelerator, etc. in the future	Moderate	4α, 2β^−^	27.9	47–85	61–230	218 and 440 keV
^227^Th	18.7 days	^227^Ac generator	High	5α, 2β^−^	32.8	50–70	71–230	84, 95, 236 and 270 keV

* Average value per decay: ^149^Tb has alpha energy of 3.97 MeV but with only 16.7% abundance.

**Table 3:** Comparisons of prostate-specific membrane antigen (PSMA)-targeting small molecules, antibodies, and their fragments [55,76,77,78,79,80].

Characteristics	Small Molecules	Antibody Fragments	Antibodies
Molecular weight	<1.5 kDa	15–110 kDa	150 kDa
Structure	*Peptidomimetic* chain	One to four polypeptide chains	Complex; four polypeptide chains
Manufacture	Easy	Difficult	Difficult
Stability	Stable	Instable	Instable
In vivo half-life	Few hours	0.5–30 h	2–7 days
Pharmacokinetics	Rapid clearance from blood, rapid tissue penetration	Rapid to intermediate clearance from blood, rapid to intermediate tissue penetration	Circulates long in blood, slow tissue penetration, longer tumor retention
Excretion	Renal clearance	Renal clearance of Ab fragments <70 kDa	Hepatobiliary clearance and Fc-receptor- mediated recycling
Target binding to the extracellular domain	Enzymatic pocket of the catalytic domain	Apical region of the extracellular domain	Apical region of the extracellular domain
Immunogenicity	Seldom	Low	Expected
Examples	PSMA-617, PSMA-I&T, NG001	IAB2M, scFvD2B, JVZ-008, PSMA6 and 30	J591, 107-1A4, PSMA-TTC

**Table 4:** Overview of α-emitting radionuclides and PSMA-targeting agents investigated in preclinical studies for targeted prostate cancer therapy. mAb, monoclonal antibody; NA, not available; Fab’, antigen-binding fragment; %ID/g, percent injected dose per gram of tissue. Therapeutic index (TI) is defined as the median survival of the treatment group divided by the median survival of the untreated group.

Radio- Nuclide	PSMA Targeting Agent	Activity	Main Observations	References
Preclinical studies in vitro				
^213^Bi	J591 (mAb)	0–1.8 MBq/mL	Antitumor activity, growth delay of LNCaP-LN3 spheroids	Ballangrud et al., 2001 [81]
^211^At	ABCPUP	NA	Binds to PC3 PIP cells	Vaidyanathan et al., 2009 [82]
^227^Ac		0–370 kBq/mL	Antitumor activity (LNCaP, Mat-Lu cells)	Bandekar et al., 2014 [83]
^223^Ra	NA-silane-PEG-D2B (mAb)	0–20 kBq/mL	Antitumor activity, LD_50_ ≈ 2.5kBq/mL (C4-2 cells)	Czerwińska et al., 2020 [84]
Preclinical studies in vivo				
^149^Tb	PSMA-617	2 × 3 MBq	37%ID/g (tumor targeting at 1 h, subcutaneous, PC3 PIP cells); antitumor activity, TI ≈ 1.8	Umbricht et al., 2019 [85]
^211^At	107-1A4 (mAb)	370 kBq	PSA decline (intratibial, C4-2B cells)	Wilbur et al., 2009 [86]
	107-1A4 Fab’	740 kBq	25%ID/g (tumor targeting at 1 h, subcutaneous, LNCaP cells)	Wilbur et al., 2011 [87]
	PSMA 6	740 kBq	14%ID/g (tumor targeting at 1 h, subcutaneous, PC3 PIP cells); antitumor activity, TI ≈ 2.1; delayed nephropathy dose limiting; dehalogenation in vivo	Kiess et al., 2016 [88]
^131^I as a surrogate for ^211^At	RPS-027	NA	Dual targeting to PSMA and albumin; 9%ID/g (tumor targeting at 1 h, subcutaneous, LNCaP cells)	Kelly et al., 2017 [89]
	16b	NA	15%ID/g (tumor targeting at 1 h, subcutaneous, C4-2B cells)	Li et al., 2020 [90]
^212^Pb/ ^212^Bi	CA009, CA012	NA	25%ID/g (tumor targeting at 1 h (^203^Pb) subcutaneous, C4-2 cells)	Dos Santos et al., 2019 [91]
	L2	1.5 and 3.7 MBq	22%ID/g (tumor targeting at 1 h (^203^Pb), subcutaneous, PC3 PIP cells); antitumor activity, TI ≈ 1.9 and TI ≈ 3	Banerjee et al., 2020 [92]
	NG001	320 kBq	22%ID/g (tumor targeting at 1 h, subcutaneous, C4-2 cells); antitumor activity, TI * ≈ 2.3	Larsen, 2019 [93]; Stenberg et al., 2020 [94,95]
^213^Bi	J591 (mAb)	3.3 MBq	Antitumor activity, TI ≈ 1.8 (subcutaneous, LNCaP cells)	McDevitt et al., 2000 [96]
		3.7 MBq	Antitumor activity, inhibition of tumor growth in mice (subcutaneous, LN3 cells)	Li et al., 2002 [97]
	PSMA I&T	5.4–6.6 MBq	5.8%ID/g (tumor targeting at 1 h, subcutaneous, LNCaP cells)	Nonnekens et al., 2017 [98]
	JVZ-008 (nanobody)	4.5–5.4 MBq	2.7%ID/g (tumor targeting at 1 h, subcutaneous, LNCaP cells)	
^225^Ac	PSMA-617	40 kBq	Antitumor activity (subcutaneous, C4-2 cells); weight loss at 100 kBq	Meyer et al., 2019 [99]
		40 kBq	Antitumor activity (intravenous, C4-2 cells), TI ≈ 3.9	Stuparu et al., 2020 [100]
		40 kBq	Antitumor activity, (subcutaneous, RM1-PSMA^+++^ cells)	Current et al., 2020 [101]
		30 kBq	Antitumor activity (subcutaneous, RM1-PGLS cells), TI ≈ 1.2	Czernin et al., 2020 [102]
	RPS-074	148 kBq	6%ID/g (tumor targeting at 4 h, subcutaneous, LNCaP cells); antitumor activity, complete response in 86%	Kelly et al., 2019 [103]
^227^Th	PSMA-TTC (mAb)	100–500 kBq/kg	20%ID/g and 37%ID/g (tumor targeting after 3 and 7 days, respectively, subcutaneous, MDA-PCa-2b cells); antitumor activity	Hammer et al., 2020 [104]

* Therapeutic index (TI) is defined as 75% survival of the treatment group divided by the 75% survival of the untreated group.

**Table 5:** Overview of α-emitting radionuclides and PSMA-targeting agents investigated in clinical studies for targeted prostate cancer therapy. *n*, number of patients; PFS, progression-free survival; OS, overall survival; -, activity de-escalation in subsequent cycles.

PSMA- TAT	*n*	Activity per Cycle	PSA Decline After TAT (Patients)		Median PFS/OS (Months)	Toxicity	References
			≤0%	≥50%			
^213^Bi- PSMA- 617	1	296 MBq		100% (1/1)	NA	NA	Sathekge et al., 2017 [105]
^225^Ac- PSMA- 617	2	100 kBq/kg		100% (2/2)	NA	Xerostomia	Kratochwil et al., 2016 [42]
	14	50–200 kBq/kg	22% (2/9)	44% (4/9)	NA/8.5	Xerostomia	Kratochwil et al., 2017 [106]
	40	100 kBq/kg	13% (5/40)	63% (24/38)	NA/>12	Xerostomia	Kratochwil et al., 2018 [107]
	1	8 MBq		100% (1/1)	NA	NA	Sathekge et al., 2019 [108]
	17	8–4 MBq	6% (1/17)	88% (15/17)	NA	Xerostomia	Sathekge et al., 2019 [109]
	1	8–6 MBq		100% (1/1)	NA	Xerostomia xero- phthalmia	De Medeiros et al., 2019 [110]
	73	8–4 MBq	18% (13/73)	70% (51/73)	15.2/18.0	NA	Sathekge et al., 2020 [111]
	26	8–4 MBq	11% (3/26)	65% (17/26)	3.5/7.7	Xerostomia, anemia, leucopenia, thrombopenia	Feuerecker et al., 2020 [112]
	28	100 kBq/kg	18% (5/28)	39% (11/28)	12/17	Transient fatigue, xerostomia	Yadav et al., 2020 [113]
	2	NA	NA	NA	NA	Chronic kidney disease	Pelletier et al., 2021[114]
	13	8–6 MBq	15% (2/13)	69% (9/13)	NA/8.5	Xerostomia	Van der Doelen et al., 2020 [115]
^225^Ac- PSMA I&T	1	8 MBq		100% (1/1)	NA	Xerostomia	Ilhan et al., 2020 [116]
	14	7.8 MBq	21% (3/14)	50% (7/14)	NA	Xerostomia	Zacherl etal, 2020 [117]

**Table 6:** Ongoing clinical trials with targeted α therapies (TAT) targeting PSMA in metastatic castration-resistant prostate cancer (mCRPC) patients registered in https://clinicaltrials.gov/ (accessed on 14 January 2021).

Trial ID	Phase	TAT	Number of Patients	Period	Sponsor
NCT03276572	1	^225^Ac-J591	42	2017–2021	Weill Medical College of Cornell University
NCT04506567	1/2	^225^Ac-J591	105	2020–2025	Weill Medical College of Cornell University
NCT04225910	1	^225^Ac-PSMA	20	2019–2021	Xinhua Hospital, Shanghai Jiao Tong University School of Medicine
NCT04597411	1	^225^Ac-PSMA-617	30	2021–2022	Novartis Pharmaceuticals
NCT03724747	1	BAY 2315497 (^227^Th-mAb)	157	2018–2023	Bayer

**Table 7:** PSMA expression in prostate cancer cell lines. N/A, not applicable; negative, below the lower limit of quantification.

Cell Line	Number of PSMA Per Cell	References
DU145	Negative	[199,200]
PC3	Negative	[199,200]
22Rv1	15,000	[101]
RM1-h-PSMA	19,000	[201]
LS174T-PSMA	43,000	[138]
PSMA^++^ RM1	49,000	[101]
RM1-PGLS	56,000	[201]
MDA PCa2b	118,000	[200]
LNCaP	126,000–250,000	[96,138,199]
C4-2	102,000–255,000	[101,199,201]
PC3 PIP	552,000	[101]
